# 4580 Development of a play-based coaching intervention to improve quality of life and wellbeing for mothers with cancer and their young children

**DOI:** 10.1017/cts.2020.121

**Published:** 2020-07-29

**Authors:** Rosa Roman-Oyola, Anita Bundy, Eida Castro, Osiris Castrillo

**Affiliations:** 1 University of Puerto Rico- Medical Sciences Campus

## Abstract

OBJECTIVES/GOALS: Mothers with cancer who have young children experience life disruptions when treatment procedures limit mother-child interactions. This study proposes the development of an intervention combining the Coaching approach with the Model of Playfulness to improve Quality of Life (QoL) and wellbeing of these patients and their young children. METHODS/STUDY POPULATION: This embedded mixed method study will be guided by the two initial phases of the ORBIT Model for the development of behavioral interventions for patients with chronic diseases. Participants will be mothers in the post-acute treatment stage of cancer (n = 6) and their children who are between 2 years and a half and 6 years, 11 months. Phase 1A, Definition, builds on qualitative data from a concurrent study exploring the experiences of mothers with cancer playing with their young children. As part of this phase, we will develop a play-based coaching intervention. In Phase 1B, Refinement, we will employ in-depth semi-structured interviews and standardized tools to evaluate acceptability of the intervention and preliminary outcomes. This will serve to further refine the intervention. RESULTS/ANTICIPATED RESULTS: Phase 1A will yield a plan for the intervention and data to enhance its initial implementation. Phase 1B will yield data, from the perspective of the mothers, about acceptability of the intervention procedures (e.g., delivery strategy, place for the intervention, time devoted, and outcome measures). This will enable modifications to the intervention. Additionally, Phase IB will yield preliminary data from specific QoL and wellbeing measures. For the mother, data about anxiety and depression symptoms, stress levels, and parental self-efficacy; for the child, emotional and behavioral indicators; for both: playfulness. DISCUSSION/SIGNIFICANCE OF IMPACT: This study entails the development of an intervention to enhance QoL and wellbeing of mothers with cancer and their children. Play moments as the centerpiece of the intervention, represent an innovative approach. Findings will guide the design of future feasibility studies to advance the development of this outcome driven intervention.

